# Oncolytic Newcastle disease virus delivered by Mesenchymal stem cells-engineered system enhances the therapeutic effects altering tumor microenvironment

**DOI:** 10.1186/s12985-020-01326-w

**Published:** 2020-05-05

**Authors:** Mohsen Keshavarz, Mir Saeed Ebrahimzadeh, Seyed Mohammad Miri, Hassan Dianat-Moghadam, Seyedeh Sara Ghorbanhosseini, Seyed Reza Mohebbi, Hossein Keyvani, Amir Ghaemi

**Affiliations:** 1 The Persian Gulf Tropical Medicine Research Center, The Persian Gulf Biomedical Sciences Research Institute, Bushehr University of Medical Sciences, Bushehr, Iran; 2 Department of Medical Virology, School of Medicine, Iran University of Medical Sciences, Tehran, Iran; 3 Department of Microbiology, Golestan University of Medical Sciences, Gorgan, Iran; 4 Department of Chemistry, Sharif University of Technology, Tehran, Iran; 5 Stem Cell Research Center, Tabriz University of Medical Science, Tabriz, Iran; 6 Department of Clinical Biochemistry, Faculty of Pharmacy, Isfahan University of Medical sciences, Isfahan, Iran; 7 Gastroenterology and Liver Diseases Research Center, Research Institute for Gastroenterology and Liver Diseases, Shahid Beheshti University of Medical Sciences, Tehran, Iran; 8 Department of Virology, Pasteur Institute of Iran, Tehran, Iran

**Keywords:** Mesenchymal stem cells, Oncolytic Newcastle disease virus, Human papillomavirus, CD8 +, Tumor microenvironment, Myeloid-derived suppressor cells

## Abstract

**Background:**

Human papillomavirus (HPV)-associated malignancy remain a main cause of cancer in men and women. Cancer immunotherapy has represented great potential as a new promising cancer therapeutic approach. Here, we report Mesenchymal stem cells (MSCs) as a carrier for the delivery of oncolytic Newcastle disease virus (NDV) for the treatment of HPV-associated tumor.

**Methods:**

For this purpose, MSCs obtained from the bone marrow of C57BL mice, then cultured and characterized subsequently by the flow cytometry analysis for the presence of cell surface markers. In this study, we sought out to determine the impacts of MSCs loaded with oncolytic NDV on splenic T cell and cytokine immune responses, caspase-3 and -9 expression, and myeloid and myeloid-derived suppressor cells (MDSCs) by histological and immunohistochemical studies in the tumor microenvironment (TME).

**Results:**

Our findings proved that MSCs possess both migratory capacity and tumor tropism toward transplanted tumor tissue after peritumoral administration. Tumor therapy experiments indicated that oncolytic NDV delivered by MSCs-engineered system significantly reduces tumor growth, which is associated with the enhancement of E7-specific lymphocyte proliferation, CD8+ T cell cytolysis responses, and splenic IFN-γ, IL-4 and IL-12 responses compared with control groups. Moreover, the treatment upregulated the concentration of apoptotic proteins (caspase 9) and increased infiltration of tumor microenvironment with CD11b + myeloid and Gr1 + MDSCs cells.

**Conclusions:**

Our data suggest MSCs carrying oncolytic NDV as a potentially effective strategy for cancer immunotherapy through inducing splenic Th1 immune responses and apoptosis in the tumor microenvironment.

## Background

Human papillomavirus (HPV) is one of the most usual reproductive tract viral infection that accounts for approximately 90% of cervical and anal carcinomas and also 60% of oropharyngeal cancer [1]. Human papillomavirus (HPV) 16 and 18 are two types of high-risk HPV associated with most malignancy. The growth of HPV-associated cancers depends on the continued expression of the viral E6 and E7 oncogenes [2]. In spite of advances in diagnostic methods, these types of cancer are reported to cause 640,000 new cases annually in both sexes [3]. Thus, developing novel therapeutic approaches is urgently in demand [4, 5].

Cancer cells with high replicative activity, their deficiencies in antiviral type I interferon signaling, and cell surface overexpression of receptors-mediated cellular entry of virus [6], all provide the opportunity to employ oncolytic viruses (OVs) as a novel tool for cancer therapy. OVs can selectively infect and kill tumor cells while leaving normal cells intact [7].

The principal systemic anti-tumor mechanism of oncolytic viruses is likely based on the virally induction of immune response to identify and present tumor antigens. Oncolytic virus replication within tumor cells attracts immune cells into the tumor microenvironment, leading to cross-priming of tumor-associated antigens (TAAs) for triggering the effective antitumor immunity [8, 9].

Newcastle disease virus (NDV) is an RNA virus belonging to the paramyxovirus family and has been known to induce apoptotic cell death in malignant cell lines [10]. As an oncolytic virus, NDV is a potential tool for cancer therapy and has proved to be a safe and effective antitumor agent [11].

It has been shown that the NDV prevails the immunosuppressive micro-environment of tumors and can directly lead to promoted immune responses due to the increased production of cytokines, particularly interferon (IFN) and the immunogenic cancer cell death (ICD) [12].

One of the major obstacles in the use of naked viruses in cancer virotherapy is the host immune system, which reduces the effectiveness of treatment through complement-mediated antibody-dependent neutralization [13].

To protect the oncolytic virus from the adverse effects of immune-mediated clearance or neutralization, using cell carriers have been proposed as a novel approach [13, 14]. In addition, the use of cells with intrinsic capability to migrate within the tumor microenvironment for delivery of anti-cancer agents increases the effectiveness of treatment [15]. Previous studies have evaluated several candidates of carrier cells, including monocytic cells, dendritic cells, mesenchymal stem cells (MSCs), and tumor cells [16]. MSCs represent the characteristics of a promising delivery vehicle that protect the oncolytic viruses from the effects of complement-mediated neutralizing antibodies [12], and also possess the unique ability to steer them toward inflammation and tumor growth sites [14].

The use of MSCs to deliver the oncolytic measles virus is underway in phase I/II clinical trial (NCT02068794). It has been demonstrated that by employment of the carrier cell, the virus particles escape detection by the circulatory system and evade the immune system. Furthermore, due to the tumor-homing abilities and also their suitability for virus replication, these delivery vehicles can be used for therapeutic purposes [15]. Considering these, we aimed at assessing the in vivo efficiency of cancer immunotherapy on a syngeneic murine papillomavirus cancer model using MSCs loaded with NDV.

## Methods

### Virus and cell lines

The LaSota NDV strain used in this study was prepared from Razi Institute of Serum and Vaccine Research Center. The strain was propagated in the allantoic cavity of 9- to 11-day-old SPF embryonated chicken eggs, and all allantoic fluid samples were harvested and kept at − 80 °C until use. The titer of the virus was determined using Embryo Infectious Dose 50 (EID50). In order to inactivate NDV, the sample was exposed to the UV radiation [17] and the result was confirmed by Vero cell line [18, 19]. The finding revealed that the UV-inactivated NDV does not create any plaques in Vero cells. EID50 is commonly used as a titration unit of the NDV. For EID50 to PFU conversion, we used EID/50 ∼ 0.7 PFU formula [20].

The murine TC-1 cell line was purchased from the National Cell Bank of Iran (Pasteur Institute of Iran). Briefly, TC-1 cells were cultured in complete RPMI 1640 media (Gibco BRL, Gaithersburg, MD, USA) containing 10% fetal bovine serum (FBS) (Gibco, Rockville, MD), 100 U/mL of penicillin, 100 μg/mL of streptomycin and 0.4 mg/mL G418 (all from GIBCO, UK), 0.5 mM sodium pyruvate (Sigma Aldrich, Germany), and 2 mM L-glutamine. The EL4 cell line (murine T-cell lymphoma of haplotype H-2b derived from C57BL/6 mice) was cultured in RPMI 1640 supplemented with 10% FBS. In addition, MSC cells were flushed from the femurs and tibia of female 6–8-week-old C57BL/6 mice and seeded onto a petri dish containing MSC DMEM F-12 medium (Gibco, UK), 10% FBS, and 100 U/mL of penicillin, 100 μg/mL of streptomycin. The cells were grown for 2–3 weeks until almost confluent. Adherent cells were then detached by 0.25% trypsin-EDTA and replated using a 1:3 dilution until the third passage. All the cells were incubated at 37^∘^C in a humidified 5% CO_2_ incubator.

### Mice

Six- to 8-week-old female C57BL/6 (H2b) mice were purchased from the Institute Pasture of Iran (Tehran, Iran). The mice were adapted to the environment for 1 week before the experiment, and had free access to food and water and were kept in a 12–12 light period. All experiments were performed according to the Ethical Committee for the use and care of laboratory animals of Iran University of Medical Sciences (ethics number: IR.IUMS.FMD.REC 1396.9321540001).

### Flow cytometry analysis

To verify the stromal nature of cultured cells, the expressions of surface antigens CD44 and CD105 as MSCs markers and antigens CD45 and CD34 as hematopoietic cell markers with proper control isotypes and flow cytometry (BD FACS ARIA II, Becton Dickinson, San Jose, CA, USA) were assessed following fluorochrome-conjugated monoclonal antibodies, including fluorescein isothiocyanate (FITC)-labeled anti-CD44 (Cat. No. 561859) and -CD105 (Cat. No. 565944) (50 μg/ml), and phycoerythrin (PE)-labeled anti-CD45 (Cat. No. 561087) and -CD34 (Cat. No. 551387) (25 μg/ml) (all from BD Biosciences, USA). Briefly, cultured cells were trypsinized with 2.5% trypsin-EDTA, washed twice with PBS, and incubated with 10 μl of mentioned antibodies at room temperature for 30 min in the dark. Permeabilization with 0.1% Triton X-100/PBS for 1 min was performed before incubation with the intracellular markers. The cell populations were then characterized according to the surface markers using a FACS Calibur flow cytometer (BD Bioscience, USA). The data were collected and analyzed using Flowjo software (Version 7.6). In addition, nonspecifically labeled proteins were identified by suitable isotype-matched antibodies.

### In vitro osteogenic and adipogenic differentiation potential of MSC

Bone marrow MSCs (BM-MSCs) at third passage had been cultured in 12-well cell culture plate (SPL Inc., Korea) until approximately 90% confluence before adipogenic and osteogenic differentiation media were added as previously described [21]. Adipogenic differentiation medium was made with DMEM supplemented with 10 μg/ml insulin and 10^− 6^ M dexamethasone (all supplements from Sigma, St Louis, MO). Adipogenesis was confirmed after 21 days by Oil Red O (Sigma-Aldrich) staining and the accumulation of neutral lipids in fat vacuoles.

Osteogenic differentiation medium was made with DMEM supplemented with 10^− 8^ M dexamethasone, 10 mM β-glycerophosphate, and 50 μg/ml ascorbic acid (all supplements from Sigma, St Louis, MO). The media was changed twice a week for 3 weeks. Matrix mineralization was confirmed by calcium phosphate deposits after staining with 0.2% Alizarin Red S (2% aqueous solution, pH 4.1–4.3, adjusted with ammonium hydroxide) for 20 min.

### Viral infection of BM-MSCs with oncolytic NDV

The isolated MSCs were cultured in a six-well plate at a cell density of 5 × 10^5^ cells/well for 24 h. To determine the packaging efficiency of the MSCs, the MSCs were infected with the various multiplicity of infection (MOI) (1–40) of NDV for 1 h in DMEM F-12 medium. Afterward, NDV-encapsulated MSCs were washed with PBS for removal of the NDV from the supernatant medium. The cytotoxic effects of the different NDV MOIs on MSCs was evaluated by Cell viability assay (MTT) (sigma, USA) [22, 23].

### In vivo tumor treatment experiment

In vivo tumor induction was conducted through subcutaneous (s.c.) injection of 7 × 10^5^ TC-1 tumor cells/mouse into the right flank area of the mice on day 0, then they randomly divided into six different groups (10 mice/group). Ten days after tumor cells injection, C57BL/6 mice were treated peritumorally (p.t.) with MSC (10^5^ cell/100 μl), MSC/iNDV (10^5^ cell/100 μl containing inactivated NDV), MSC/NDV (10^5^ cell/100 μl containing activated NDV), iNDV (10^8^ PFU/100 μl inactivated NDV), NDV (10^8^ PFU/100 μl activated NDV) and PBS (100 μl) twice at 1 week intervals. Tumor growth and survival were monitored two to three times a week. Thereafter, mice were monitored twice a week by inspection and palpation. Tumor size was monitored by measuring the length (i.e., the longest dimension) and width (i.e., the shortest dimension) using electronic calipers. Tumor volume was calculated by the following formula: tumor volume = 0.5 × (length + width^2^).

### BM-MSC transduction and GFP reporter gene detection

BM-MSCs were transduced with a lentiviral vector expressing the enhanced green fluorescent protein (eGFP) gene (a gift from Stem Cell Technology Research Center, Tehran, Iran) at a multiplicity of infection of 10 (MOI = 10) and the transduction efficiency was evaluated directly in cell culture using fluorescence microscopy (Olympus, Tokyo, Japan) after 24 h (Fig. 2).

In order to track the migration and distribution of injected BM-MSCs transduced with eGFP (MSC-eGFP) in the tumor microenvironment, 10^5^ MSCs at the third passage in 100 μL of PBS were injected into tumor-bearing mice through the peritumoral administration procedure. Mice (*n* = 3/group) were sacrificed under deep anesthesia after the injection, and the intensity of fluorescent signal was evaluated in tumor sections.

### Lymphocyte proliferation assay (LPA)

In order to investigate whether treatment with the mesenchymal stem cells infected with oncolytic NDV could induce antigen-specific cell-mediated immunity, lymphocyte proliferation was performed in vitro. In this assay, the capability of re-stimulated splenocytes in converting tetrazolium to insoluble purple formazan was evaluated. One week after last treatment, splenocyte culture at 2 × 10^5^ cells/well was established in 96-well round-bottom plates containing RPMI-1640 supplemented with 10% FBS, 1% L-glutamine, 1% HEPES, and 0.1% penicillin/streptomycin (in triplicate), followed by 72 h incubation at 37 °C in a 5% CO_2_ incubator in the presence of 1 μg/ml E7-specific H-2Db CTL epitope (1 μg/ml, Biomatik, Ontario, Canada, > 99% purity), PHA (positive control), and medium alone as negative control. Afterward, the supernatants were removed, and the pellets were solubilized in 100 μl dimethyl sulfoxide attempting to eliminate the possibly produced crystals of formazan. Plates were read at a wavelength of 540 nm and stimulation index was used for expressing the results. This index was obtained as follows:

$$ \mathrm{stimulation}\ \mathrm{index}=\frac{\mathrm{OD}\ \mathrm{values}\ \mathrm{of}\ \mathrm{stimulated}\ \mathrm{cell}\mathrm{s}\ \left(\mathrm{Cs}\right)-\mathrm{relative}\ \mathrm{cell}\ \mathrm{numbers}\ \mathrm{of}\ \mathrm{unstimulated}\ \mathrm{cell}\mathrm{s}\ \left(\mathrm{Cu}\right)}{\mathrm{relative}\ \mathrm{OD}\ \mathrm{values}\ \mathrm{of}\ \mathrm{unstimulated}\ \mathrm{cell}\mathrm{s}} $$

### In vitro cytotoxic activity

To confirm whether mesenchymal stem cells infected with oncolytic NDV could induce cytotoxic immune responses by activating antigen-specific cytotoxic T lymphocytes, in vivo cytotoxic T lymphocyte (CTL) assay was performed by the measurement of lactate dehydrogenase (LDH) release. One week after the last treatment, a single-cell suspension of splenocytes was prepared and applied as effector cells. For the preparation of the target cells, EL4 cells were pulsed with 1 μg/ml E7-specific H-2Db CTL epitope. An exact viable number of 4 × 10^4^ EL4 cells in a volume of 100 μl (as target cells) were co-cultured with effector cells (100 μl) at 50:1 effector-to-target cell (E/T) ratios, in which a maximal release of LDH was observed. After centrifugation, the supernatants (50 μl/well) were transferred to 96-well plates, and CTL activity was measured [24].

### Cytokine ELISA assay

Seven days after the second treatment, the spleen of the mice (*n* = 3) were isolated and mononuclear cells from spleen of immunized mice were seeded at a concentration of 2 × 10^5^ cells/well in 24-well plates (TPP, Switzerland) for 3 days in RPMI1640 supplemented with 10% FBS, 1% L-glutamine, 1% HEPES, 2.5 mM 2-mercaptoethanol, and stimulated with E7-specific H-2Db CTL epitope at a concentration of 1 μg/ml (Biomatik, Ontario, Canada, > 99% purity) at 37 °C in 5% CO_2_. The cell supernatants were collected after 48 h and the secretion of IL-4, IFN-γ and IL-12 in the supernatant were evaluated by commercially available ELISA kits (R&D Systems Inc., Minneapolis, Minn, USA) following the manufacturer’s instructions. All samples were performed in triplicate and the plates were read at optical density (OD) 450 nm.

### Intratumoral activity assay of Caspase 3 and Caspase 9

Intrinsic apoptosis is one of the pathways that may be induced by oncolytic NDV. Caspase-3 and -9 activities in the tumor microenvironment were measured by caspase ELISA kit (Abcam, Cambridge, MA, USA). Briefly, the tumor tissue was extracted from each group (*n* = 3) and 100 mg of discarded tissue homogenized in 0.5 ml lysis buffer (0.1 M Tris-HCl (pH 7.6) and 0.1 M fresh dithiothreitol). After centrifugation at 10,000×g (1 min), equal amount of supernatant was added to the substrate-containing reaction buffer (0.1 M dithiothreitol and 5 μl of 4 mM DEVD-p-NA) and incubated for 120 min at 37 °C. Finally, the caspase-9 and -3 activities were assessed by the microplate reader (BioTek, 800TS, USA) at an absorbance of 405 nm. Each experiment was repeated in triplicate.

### Histology and immunohistochemistry (IHC)

For histological analysis, harvested tumor tissues were collected and immersed in 10% buffered formalin and then embedded in paraffin. Specimens were sectioned at 5 μm thickness and stained with hematoxylin/eosin (H&E). Finally, all specimens were observed under microscope (Nikon) and images were captured with digital camera (RT color SPOT). Subsequently, mitotic cells and histological structure between different groups were compared.

To evaluate the level of myeloid and myeloid-derived suppressor cells (MDSCs) in tumor tissue, the tumor sections were also analyzed immunohistochemically using anti CD11b (BioLegend Cat. No. 101207) and anti-Gr-1 (BioLegend Cat. No. 108407) antibodies, respectively.

Briefly, Tumor tissue sections were deparaffinized and rehydrated in alcohol gradients and then washed and boiled for antigen retrieval (10 min at 95 °C). In the next step, sections were blocked using bovine serum albumin (BSA) and incubated with biotinylated goat anti-rat secondary antibody (1:500, Sigma Aldrich) for 1 h at room temperature. After washing and incubation with horseradish peroxidase (HRP)-conjugated streptavidin (Sigma-Aldrich, Pro. No. 18–152), the reaction was revealed with DAB (Sigma-Aldrich). Cell counting was performed on randomly taken photographs of IHC-stained sections from four independent samples, using an oil-immersion 100x objective. Finally, image J software (NIH, Bethesda, USA) was used to quantify stained regions.

### Statistical analysis

All statistical analysis was performed using the SPSS 16.0 software through one-way ANOVA technique. A value of *P < 0.05, **P < 0.01 and ***P < 0.001 were considered to demonstrate statistical significance.

## Results

### MSC characterization and cytotoxicity assay

**Fig. 1:**
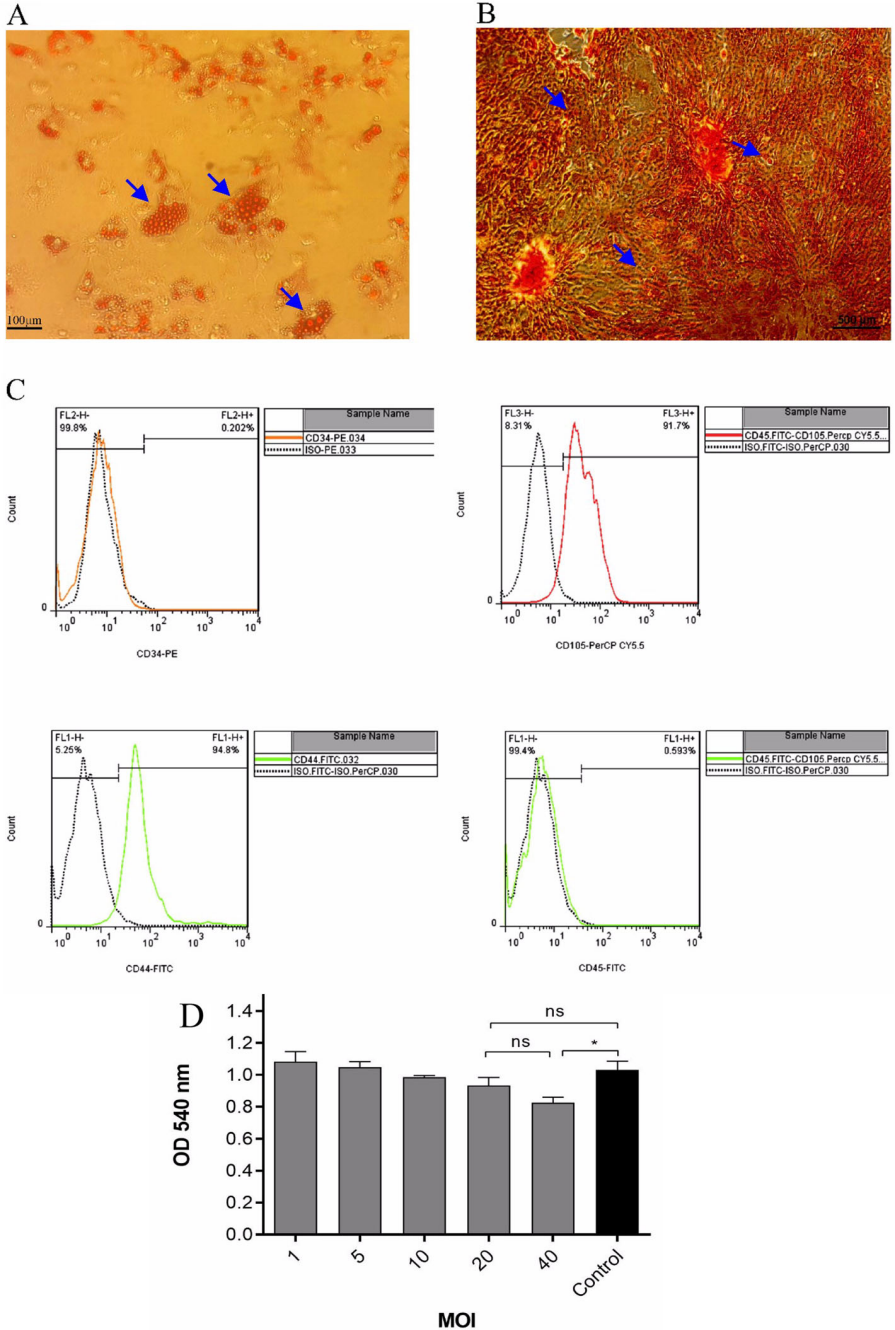
Differentiation and characterization of bone marrow mesenchymal stem cells (BM-MSCs). MSCs were isolated from the bone marrow of 6-8 week-old C57BL/6 mice by selective adherence to the plastic cell culture dishes. **a** Adipogenic (see black arrow) and **b** osteogenic (see white arrow) differentiation were evaluated by Oil Red O staining and Alizarin Red as described in the materials and methods. **c** Flow cytometry quantification of MSCs after isolation and enrichment showed the MSCs were positive for CD44 and CD105 but negative for CD34 and CD45 markers. **d** The cytotoxic effects of the different NDV MOIs on MSCs was evaluated by MTT assay at 72 h post-infection. Means are from 3 independent experiments and *(*P* < 0.05) indicates statistically significant difference between MOI 40 compared with control group

The proliferation of the bone marrow-derived mesenchymal stem cells of female mice femur as a hallmark of MSCs, was demonstrated through identification of Osteogenic and adipogenic differentiation of MSCs using Alizarin Red and Oil Red O staining, respectively (Fig. 1a, b). These findings indicated that obtained cells were able to differentiate into osteogenic (calcium phosphate deposition) and adipogenic (lipid granules accumulation) cells. Furthermore, flow cytometric analysis was performed on cultured cells at the third passage to characterize the purity of MSCs using markers commonly used to characterize mouse mesenchymal cell surface markers, namely, CD45, CD34, CD105, and CD44 antibodies. The analyses showed that the majority of BM-MSCs expressed high levels of CD44 (94.8%) and CD105 (91.7%) cell surface markers. Although, the majority of BM-MSCs were negative for CD45 (99.4%), a hematopoietic cell surface marker; and CD34 (99.8%) (Fig. 1c). This observation was consistent with the general description of the phenotypic profile of classical MSCs. In this regard, cytotoxicity results indicated that MOIs 1–20 induced only a small degree of cell death when compared with control group, although this relationship was not significant (*p* > 0.05). In contrast, we found that infection of the MSCs with 40 MOI of NDV induced significant cell death after 72 h (around 20%) (Fig. 1d). Based on the results, the MOI of 20 was chosen as an optimum dose for BM-MSCs infection. The data reported are representative of three independent experiments, each performed in duplicates.

### In vivo tracking of MSCs in tumor microenvironment by immunofluorescence microscopy

**Fig. 2:**
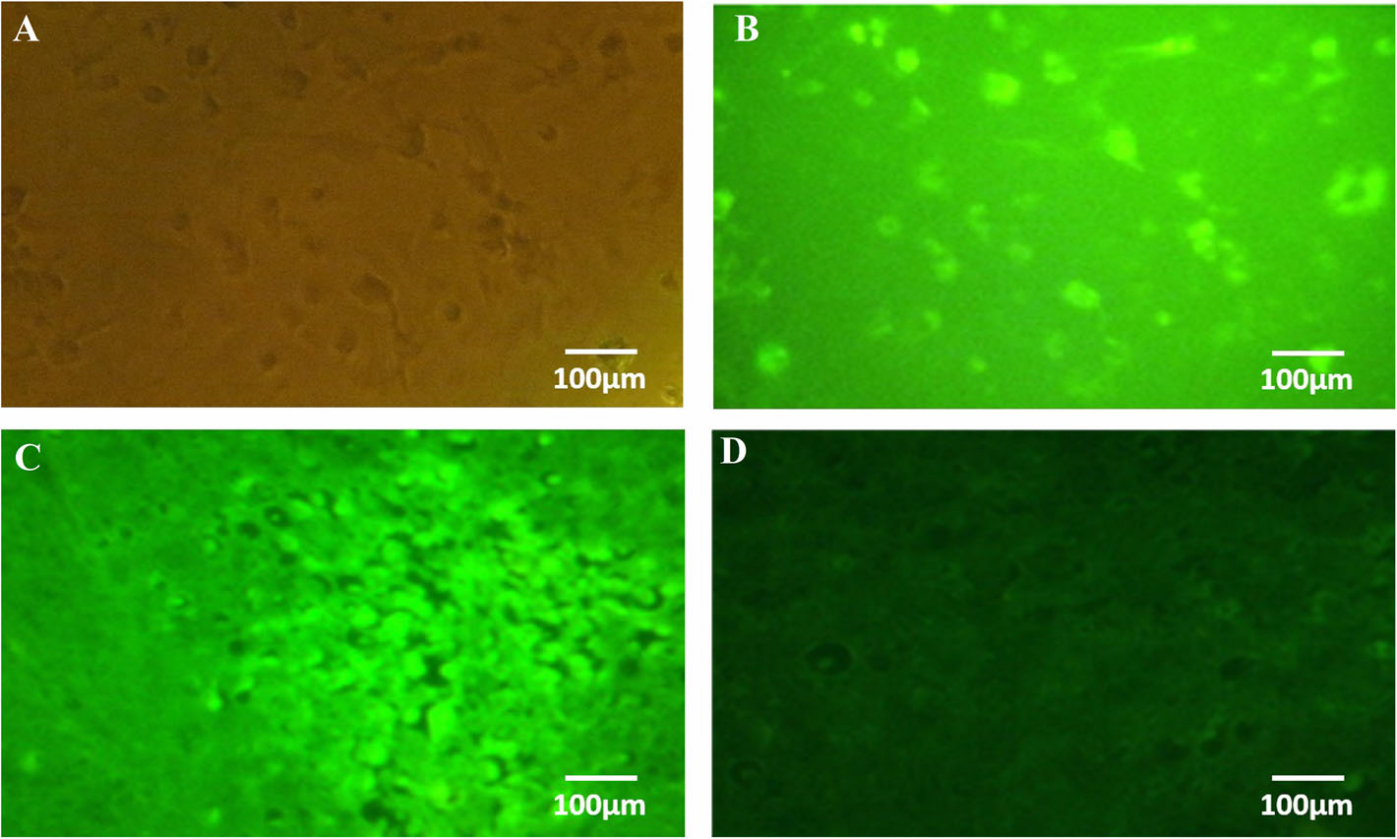
In vitro and in vivo immunofluorescence visualization of BM-MSCs. **a-b** Fluorescence microscopy of BM-MSCs transduced with lentivirus particles in vitro (40× magnification) **c** Fluorescence microscopy of BM-MSCs transduced with lentivirus particles in tumor tissue (40× magnification), **d** Fluorescence microscopy of non- transduced MSC

We investigated the tropism of MSCs to tumors in a HPV16 E6/E7+ syngeneic TC-1 mouse tumor model. For this regard, mouse MSCs transfected with lentiviral vectors expressing eGFP for the purpose of tracking migration. Transduced MSCs were administrated into tumor-bearing mice through the peritumoral route, and MSC localization was evaluated by fluorescent microscopy (Fig. 2). Our results confirmed the migratory capacity of MSCs toward TC-1 tumor after peritumoral delivery which could be detected in tumor sections on day 4 (Fig. 2-C).

### NDV-loaded MSC and NDV groups enhanced the stimulation of T lymphocytes proliferation and cytotoxic effect

**Fig. 3:**
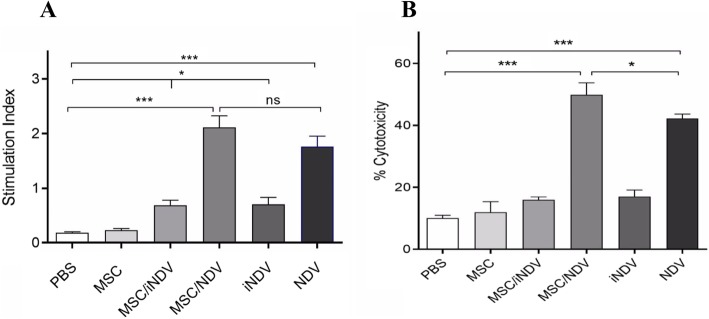
Lymphocyte proliferation assay and cytotoxic T lymphocyte response following treatment with MSCs loaded with oncolytic NDV. **a** The results indicate that MSC/NDV and NDV groups stimulate significant differences when compared with MSC/iNDV, iNDV (*P* < 0.001), PBS and MSC control groups (*P* < 0.001). Also, the stimulation index was significant in MSC/iNDV and iNDV treated groups compared to MSC and PBS control groups (*P* < 0.05). **b** Results reveal that treatment with MSC/NDV and NDV groups can significantly increase CTL responses compared with control groups (MSC and PBS groups) (P < 0.001). Also, cytolytic analysis of CTL lymphocytes demonstrated a greatly increased CD8+ activity in the group of mice treated with MSC/NDV compared with the NDV group (*p* < 0.05). The results are representative of three independent experiments. * *P* < 0.05; ** *P* < 0.01; *** *P* < 0.001

To determine whether the E7-specific lymphoproliferative response mainly resulted from a response to the BM-MSCs transduced with oncolytic NDV, Lymphocyte proliferation assay was performed in experimental groups. As previously mentioned, our treatment groups including MSC, MSC/iNDV, MSC/NDV, iNDV, NDV and PBS were treated peritumoral in two times at seven-day intervals. The mice treated with MSC/NDV and NDV showed a significant lymphocyte proliferation response when compared with the MSC/iNDV and iNDV groups (*P* < 0.001). Moreover, there was no significant difference between MSC/NDV and NDV groups (*P* > 0.05). Of note, a significant difference was observed between the MSC/iNDV and iNDV groups in comparison to the MSC and PBS control groups (*P* < 0.05). Additionally, there was no expansion of splenocytes against E7 antigen from C57BL/6 mice treated with MSC and PBS control groups (P > 0.05) (Fig. 3a). These results suggest that treatment with MSC/NDV and NDV can significantly stimulate E7-specific T-cell response. To gain more insight into the anti-tumor mechanism of the MSCs carrying oncolytic NDV, we designed an LDH release assay to assess the cytotoxic activities of the CTLs induced by HPV-16 E7 epitope. Since the highest percentage of specific target lysis was detected for E7-specific CTLs at an Effector:Target (EL4) ratio of 100:1, this ratio has been selected for further analysis. Our findings (Fig. 3b) illustrated that treated mice with MSC/NDV and NDV groups can significantly induce higher antigen-specific CTL responses compared to MSC and PBS groups (*P* < 0.001). Moreover, a significantly higher E7-specific lytic activity was detected in mice treated with MSC/NDV, as compared to mice treated with NDV(*p* < 0.05) (Fig.3b). As expected, no antigen-specific cytolytic response was observed for the C57BL/6 mice groups that had been treated with MSCs and PBS (*P* > 0.05). Finally, the result revealed that MSC/NDV and NDV groups could enhance the specific cytolytic responses against TC-1 in the syngeneic model.

### Oncolytic NDV-loaded MSCs induced anti-tumoral cytokine secretion

**Fig. 4:**
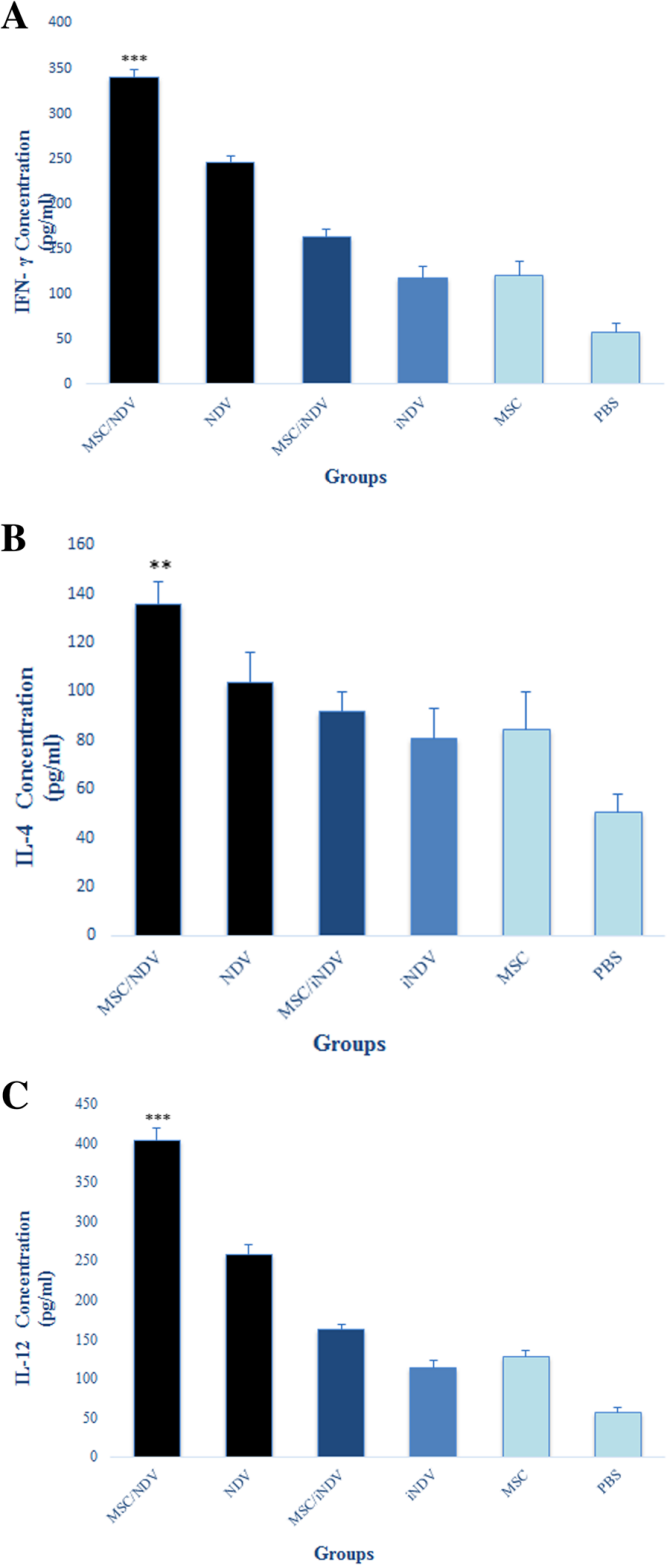
Determination of the splenic IFN-γ, IL-4, and IL-12 cytokine secretion. Two weeks after last treatment, the spleen was removed in each group(*n* = 3), seeded in 96 well plate and re-stimulated with 1 μg/ml E7-specific epitope. After 48 h, collected supernatants were assayed for the presence of IFN-γ, Il-4, and IL-12 to investigate the balance of the immune response. The level of cytokines was measured by comparison to a standard curve of serially diluted positive control samples. The data is represented as mean ± S.D. Each sample was examined in triplicate and results are representative of two experiments. * *P* < 0.05; ** *P* < 0.01; *** *P* < 0.001

Cytokine assay was employed to monitor the balance of the cellular immunity induced by MSCs carrying oncolytic NDV. As shown in Fig. 4 A, C, splenocytes from the MSC/NDV and NDV-treated mice induced a significant response of IFN-γ and IL-12 (*p* < 0.001) compared to PBS, MSC/iNDV, iNDV, and MSC groups. The production of these cytokines following the MSC/NDV treatment was significantly higher than in mice that received the NDV alone (*P* < 0.001). IL-4 levels were significantly increased when the MSC/NDV was administered compared to PBS, NDV, iNDV, MSC/iNDV and MSC groups (*P* < 0.01) (Fig. 4b). These results suggested that the MSCs carrying oncolytic NDV could induce the Th1 cytokines that may play a critical role in strengthening the anti-tumor cellular immune system.

### Screening of CD11b and gr-1 expression and histology alteration in tumor sections

The evaluation of the myeloid and myeloid-derived suppressor cells (MDSCs) was determined by the expression of CD11b + and Gr1+ markers using immunohistochemistry. We observed that treatment with MSC/NDV and NDV increased the level of CD11b + cell marker compared to MSC and PBS control groups (*p* < 0.001). Moreover, results demonstrated that the level of CD11b + marker was significant in MSC/iNDV compared to MSC and PBS control groups (*p* < 0.01). Also, the level of Gr1+ cell markers increased in MSC/NDV and NDV group compare to MSC and PBS control (*p* < 0.001). Furthermore, the level of Gr1+ increased in MSC/iNDV compared to the PBS control group (*p* < 0.01). Immunohistochemistry results indicate that treatment with MSC/NDV, NDV, and MSC/iNDV groups can induce CD11b myeloid cells which leads to an increase in the accumulation of pro-inflammatory macrophage in tumor tissue. Moreover, these treatment groups can augment MDSCs in tumor microenvironments which can be responsible for inhibitory effects on immune system cells.

**Fig. 5:**
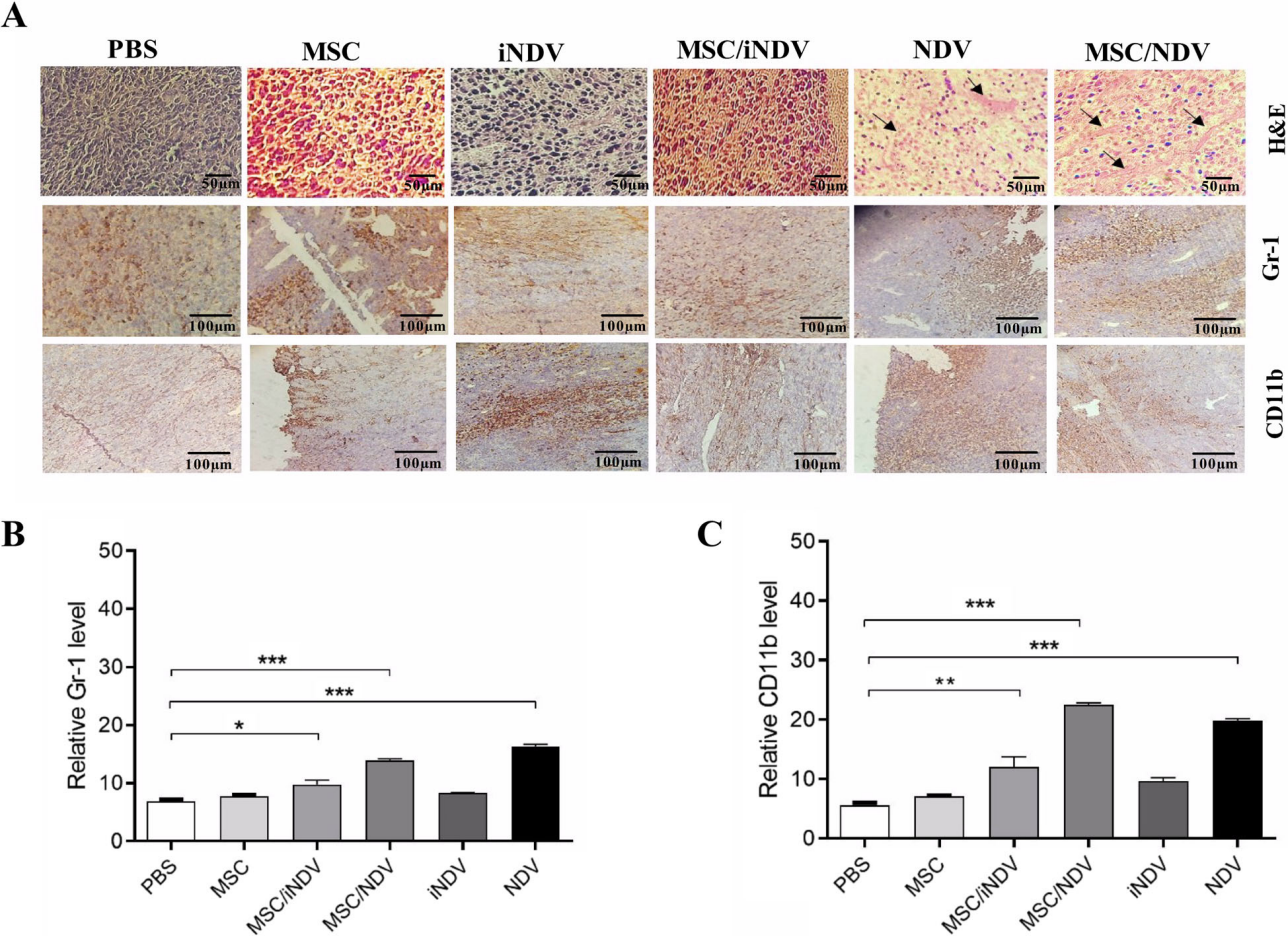
Histological and immunohistochemical variations following treatment of syngeneic TC-1 tumor model with oncolytic NDV. **a** At two weeks following final treatments, mice were sacrificed and sections of paraffin embedded or frozen tumor tissue from each group were stained with hematoxylin and eosin (H&E) for evaluation of tumor cells proliferation and signs of necrosis. Moreover, immunohistochemistry (IHC) staining was done for screening CD11b + and Gr-1+ markers. H&E, × 100 magnification, and IHC, × 40 magnification. Scale bars, 100 μm **b** The expression level of Gr-1+ in tumor sections of treatment groups. **c** The expression level of CD11b + in tumor sections of treatment groups. Immunohistochemical analysis was performed by ImageJ software. Data are expressed as means ± SEM (**b** and **c**). *n* = 3 mice per group (**b** and **c**). * *P* < 0.05; ** *P* < 0.01; *** *P* < 0.001

To illustrate the efficacy of oncolytic NDV, portions of syngeneic tumor tissue were sectioned and stained with H&E on day 7, then examined for characterization of histological differences between various treated groups. As shown in Fig. 5, H&E staining disclosed that MSC/NDV and NDV treated groups has been able to reduce the amount of proliferation of tumor cells and induced early signs of necrosis when compared to other groups particularly MSC and PBS ones.

### Oncolytic NDV induces apoptosis in vivo model

**Fig. 6:**
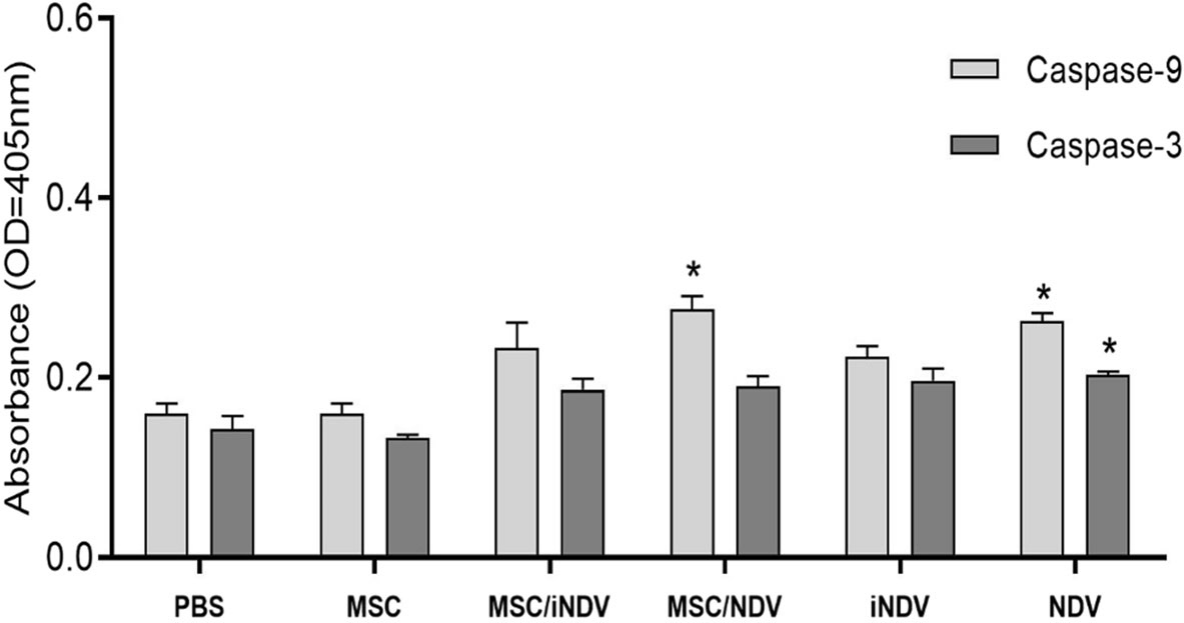
The expression level of caspase-3/9 proteins in tumor lysate of treated groups. Treatment with oncolytic NDV significantly (*P < 0.05) increased the level of Caspase-9 in NDV and MSC/NDV groups compared to control groups (PBS and MSC). Also, treatments with oncolytic NDV significantly (*p* < 0.05) increase the Caspase-3 in NDV group compared to control groups (PBS and MSC). No significant changes were observed in the levels of caspase-3 in MSC/NDV compare to control groups (PBS and MSC) (*p* > 0.05)

OVs directly induce a cytolytic effect on tumor cells or indirectly promote tumor cells apoptosis [25]. Our previous studies have shown that oncolytic NDV induces apoptosis in TC-1 cell line [26]. In addition, Kumar et al. found that NDV mediates tumor cell killing through promoting the TNF-related apoptosis-inducing ligand (TRAIL)/TRAIL-R signaling, caspases activation, as well as apoptosis in the extrinsic pathway in HeLa cells [25]. In this regard, we investigated the effects of oncolytic NDV on the activation of caspases-9 and -3 in in vivo model. To evaluate effects of NDV treatment on tumor protection could be correlated with the tumor microenvironment factors, the expression level of caspase proteins 3 and 9 were ascertained in tumor lysates by ELISA method. We observed that oncolytic NDV induced the activation of caspase-9 in both MSC/NDV and NDV groups compared to the PBS control group (*p* < 0.05). Moreover, regarding the caspase-3 expression, the results showed an increase among the NDV group compared to PBS control group (*p* < 0.05), although no significant increase was observed in caspase-3 level in the MSC/NDV group compared to control (*p* > 0.05). In the same way, no significant difference was observed in each of caspases in tumor lysate of MSC/iNDV and iNDV groups compare to PBS control group (*p* > 0.05) (Fig. 6).

Taken together, our results indicate that the use of oncolytic NDV both in the carrier cell and NDV alone could potentially induce apoptosis through the internal pathway. However, no significant changes were observed in the levels of caspase-3 in MSC/NDV in comparison with control groups (PBS and MSC) that may be due to the occurrence of non-apoptotic pathway rather than apoptosis one, since sometimes viral infection leads to activation of other programmed cell death such as necroptosis in a caspase-8-dependent pathway.

### NDV-loaded MSCs inhibits tumor growth

Based on immunological (i.e., enhanced CTL proliferation and cytokines production) and histopathological effects (enhanced apoptotic activity and MDSCs population) (Figs. 3, 4, 5 and 6), we expected that these findings could be correlated with the inhibitory action on the tumor growth and regression. Ten days following tumor cell injection, mice were randomly separated into six groups and treated two time with NDV-loaded MSCs (infected with 20 MOI of NDV), same number of iNDV-loaded MSCs, active NDV (10^8^ PFU) and same dose of inactive NDV. As a control, we injected the similar number of MSCs and equal volumes of PBS peritumoraly. Consequently, mice were examined and the growth of the tumor was measured by caliper for 6 weeks and the effect of MSC/NDV on inhibition of tumor growth as well as reduction of tumor volume was calculated.

**Fig. 7:**
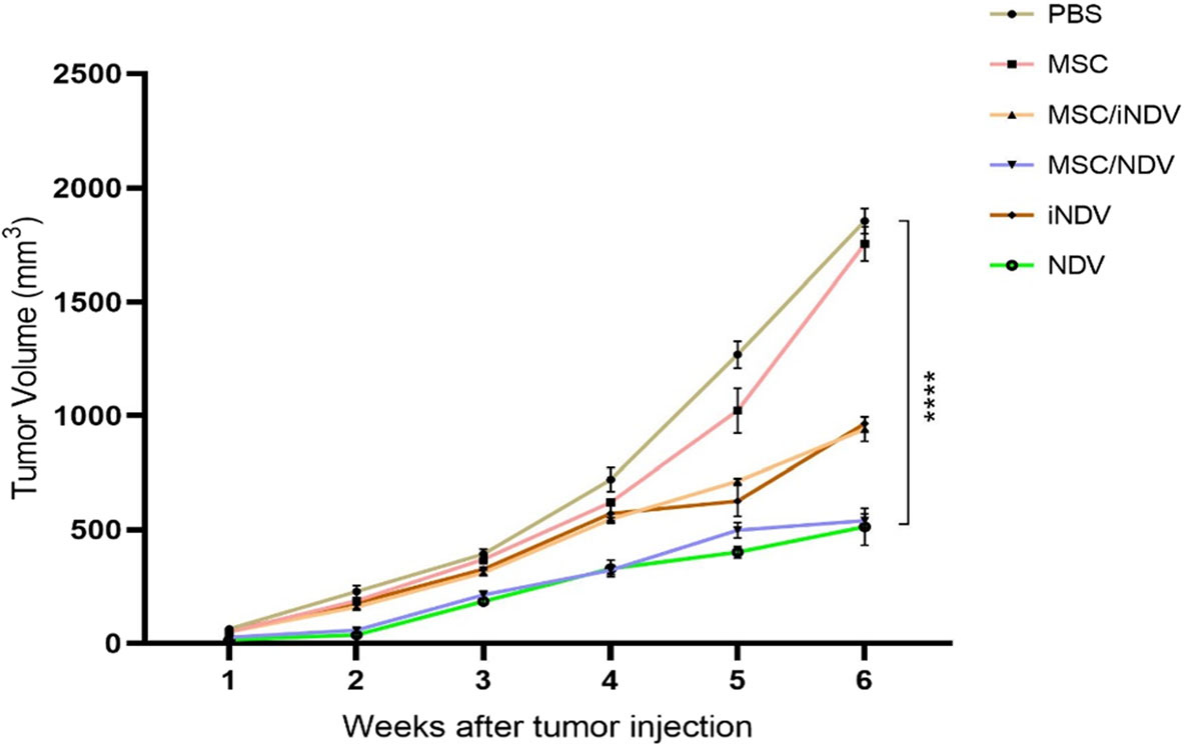
In vivo anti-tumoral response experiment in NDV-loaded MSCs mice. Tumor volume was calculated using ocular checkup and palpation for seven mice per group, three times a week during 6 weeks of monitoring. Error bars represent mean ± SD for each group of mice. ****p < 0.0001 for MSC/NDV and NDV compared to MSC and PBS groups

As expected, the results proved that in all syngeneic mouse models treated with MSC/NDV and NDV, the tumor growth significantly reduced compared to other groups (Fig. 7). No significant difference was found between the MSC/NDV and NDV groups (*p* > 0.05), which may be due to the anti tumoral effects of MSCs [27]. Cytotoxic effect of bone marrow MSCs on the established tumor in a melanoma mouse model through the release of reactive oxygen species has been presented [28]. Consistent with these results, studies showed that secretion of anti-inflammatory mediators by MSCs can lead to modulation of immune responses [29]. On the other hand, this immune modulation can be beneficial for oncolytic activity of NDV.

Moreover, the MSC/iNDV and iNDV groups could significantly control tumor growth compared to MSC and PBS control groups (*p* < 0.01) which approves the potentials of iNDV to somewhat induce apoptosis and antitumoral immune responses as shown in Figs. 3 and 6. Compared with the mild anti-tumoral effect of MSC/iNDV and iNDV groups, active NDV and NDV-loaded MSCs can remarkably reduce tumor volume by comparison with the MSC and PBS control (*p* < 0.0001). These results demonstrate that treatment with 10^8^ pfu active NDV alone or engulfed by MSC (~ 2 × 10^6^ NDV particles) promotes a strong immune response, which can turn into an anti-tumor response. Notably, the average size of the tumor was significantly reduced in MSC/NDV group compared to MSC/iNDV. In addition, on week 6, the tumor volumes of TC-1 bearing mice receiving NDV were significantly smaller than those of mice receiving iNDV alone. Together, these data confirm the hypothesis that NDV or iNDV has the potential to reduce tumor growth and its anti-tumor activity and the dose of administration are improved when loaded and delivered by MSCs as a vehicle. On the other hand, these findings indicated that the use of MSCs as vehicle has led to a optimal delivery of viral load in a reduced dose of administered oncolytic NDV (approximately 2 log). This delivery system would enable the administration of a lower viral dose that would reduce side-effects. These results also indicated that the MSCs could be used as a suitable carrier for transferring the NDV to the target site.

## Discussion

Oncolytic virotherapy is a novel method for cancer therapy which uses competent replicating viruses to selectively eliminate malignant cells [30]. However, efficient and targeted delivery of the viral-based therapy to the tumor mass remains a critical clinical challenge. The application of MSC as cell carriers for oncolytic viruses presents a novel and promising approach to overcome several barriers and augments effector function of oncolytic virotherapy in a tumor microenvironment [31]. Several studies have assessed the anti-tumoral efficacy of mesenchymal stem cells carrying oncolytic viruses for cancer therapy [31, 32].

To investigate the therapeutic effects of MSCs harboring the oncolytic Newcastle disease virus (NDV) in the human papillomavirus-associated tumor, we developed a syngeneic mouse model of papillomavirus associated cancer using immunocompetent mice. We demonstrated that MSCs as a cellular carrier efficiently migrate into the tumor tissue and deliver therapeutic oncolytic NDV. In vivo tracking of the MSCs migratory ability in the tumor microenvironment is essential for the application of stem cells for cancer immunotherapy.

Furthermore, our studies reveal that the MSCs carrying oncolytic NDV has enhanced antitumor efficacy in TC-1 tumor mouse model, which is associated with an increase in antigen-specific lymphocyte proliferation, CD8+ cytotoxicity, and IFN-γ induction. The results also indicated that the ability of MSCs carrying oncolytic NDV to induce a robust antigen-specific cytolytic immune response leads to a strong antitumor activity against E7-expressing TC-1 tumor murine model, slowing tumor growth in tumor treatment experiments in vivo. As a result, the application of MSCs carrying oncolytic NDV has been preferred due to lower undesirable systemic toxicity and efficient delivery of reduced dose of NDV.

Application of oncolytic virus for cancer therapy is currently being evaluated in clinical trials for different types of cancers. Among oncolytic viruses explored for cancer therapy, NDV has demonstrated reasonable safety profile and selective oncolysis and replication in cancer cells. In our previous study, we have confirmed the selective antitumor potential of NDV through triggering of autophagic cell death via ROS induction and activation of early apoptosis pathways, making it an encouraging virotherapeutic agent [26]. In addition to direct antitumor effect, oncolytic virotherapy also exerts a robust danger signal needed for overcoming tumor-induced immune suppression and subsequent stimulation of potent antitumor immunity in vivo [33].

Oncolytic immunotherapy has been demonstrated to release a wide range of damage-associated molecular patterns (DAMPs) and tumor-associated antigens (TAA) from whole tumor cells using oncolytic virus replication which would be taken up and cross-presented to CD8+ CTL T cells by activated dendritic cells, consequently resulting in the activation of a tumor-targeting immune response [8, 34]. In this regard, Ye et al. reported that lung cancer cells infected by NDV express a high level of several DAMPs, including HMGB1, HSP70/90, and ecto-CRT. The induction of immunogenic cell death (ICD) by oncolytic NDV can activate immune cells such as cytotoxic T lymphocytes (CTLs) and also causes the release of inflammatory responses in tumor model [35].

In support of our findings, previous oncolytic NDV findings generated from glioblastoma multiforme tumor in xenotransplant murine models have shown that virotherapy with NDV leads to enhanced infiltration of IFN-gamma+ CD4+/CD8+ T cells along with a decrease in myeloid-derived suppressor cells (MDSCs) in the tumor microenvironment [36].

A growing number of studies point out the importance of MDSCs in the regulation of immune responses in cancer and tumor progression. Meanwhile, the diverse effects of therapeutic agents on MDSC behavior have been reported [37]. Fend et al. demonstrated that employment of vaccinia virus armed with suicide gene as a tool for oncolytic virotherapy increases the infiltration of tumors by CD3 + CD8+ T lymphocytes and MDSC cells in tumor lysates of the treated group in an orthotopic model of renal carcinoma [38]. Moreover, it has been determined that administration of oncolytic HSV-1 armed with IL-12 in undifferentiated sarcoma model induces higher intra-tumoral CD8:T regulatory cell (Treg) and CD8:MDSC ratios in treated group [39]. Consistently, our findings demonstrated a pronounced infiltration of tumors with Gr1+ MDSCs in NDV, NDV/MSC and iNDV/MSC treated animals in comparison to controls. However, entering our therapy to the tumor environment may increase the local expansion of immune cells including granulocytes and monocytes. There is a possibility of a higher expression of CD11b and Gr-1 surface markers in this experiment, due to their presence of on granulocytes and monocytes.

The results also agree with recent reports about the protective properties of CD11b myeloid cells after NDV virotherapy. Myeloid cells carry out a crucial role in infection and tumor microenvironment. Integrins (such CD11b) are a family of adhesion receptors that play a key role in myeloid cells recruitment [40]. Recent studies have shown the role of CD11b integrin in anti-tumor responses and inhibition of immune suppression in animal models and human cancers [40]. Recently, Schmid et al. demonstrated that activation of CD11b leads to elevated pro-inflammatory macrophage polarization through induction of microRNA Let7a [41]. We also noticed that NDV and MSCs carrying oncolytic NDV treatments lead to the upregulation of caspase-3 and -9 in tumor tissue. Correspondingly, Chai et al. showed that NDV inhibited the growth of A549 tumor xenograft through activation of caspase-3 [42].

One of the main problems for the use of virotherapy as an anti-tumor agent is to avoid clearance by host antiviral antibodies [43]. One of the inherent characteristics of MSCs is the ability to implant in tumor tissue that is dependent on multiple cytokine receptors such CXCR4 and matrix metalloproteinase-2 (MMP-2) [44]. For the first time, Hamada et al. used the carrier cells to protect oncolytic viruses from antiviral immune responses. They demonstrated that adenovirus-loaded MSC can lead to effective induction of antitumoral CTL and anti-viral activity in syngeneic ovarian tumor model [45].

In a recent study, deployment of an oncolytic adenovirus-loaded menstrual blood-derived mesenchymal stem cells (MenSCs) vehicle enhanced antitumor responses following T and NK cells activation [46]. Another study has also documented that intratumoral injections (i.t.) of oncolytic human adenovirus-loaded mesenchymal stem cells leads to an increase in anti-tumoral T cells [15], suggesting that MSCs are an attractive vehicle for targeted delivery in oncolytic therapy. Future investigations should address whether this method has the potential for translation into the clinical applications.

## Conclusions

To sum up, our results suggest that the MSC carrier represents a valuable tool for delivery of oncolytic NDV as it induces an effective specific immune response involving T cell response, slight rise in MDSCs, and elicited significant increase in expression of caspase-9. Collectively, our findings provide the rationale for the development of oncolytic NDV-loaded MSC carrier for enhancement of treatment efficacy in HPV infection and HPV-associated diseases.

## Data Availability

The datasets used and analysed during the current study are available from the corresponding author on reasonable request.

## Abbreviations

CC: Cervical cancer
HPV: Human papillomavirus
OVs: oncolytic viruses
TAA: Tumor-associated antigens
NDV: Newcastle disease virus
IFN: Interferon
ICD: Immunogenic cancer cell death
MSCs: Mesenchymal stem cells
EID50: Embryo infectious dose 50
PFU: Plaque forming units
FITC: Fluorescein isothiocyanate
MOI: Multiplicity of infection
iNDV: Inactivated Newcastle disease virus
EGFP: Enhanced green fluorescent protein
LPA: Lymphocyte proliferation assay
LDH: Lactate dehydrogenase
H&E: Hematoxylin and eosin
MDSC: Myeloid-derived suppressor cells
BSA: Bovine serum albumin
HPR: Horseradish peroxidase
IHC: Immunohistochemistry
TRAIL: TNF-related apoptosis inducing ligand
DAMPs: Damage-associated molecular patterns
CTL: Cytotoxic T lymphocyte
MMP2: Matrix metalloproteinase-2
MenSCs: Menstrual blood-derived mesenchymal stem cells
