# Correction to: Oncolytic Newcastle disease virus delivered by Mesenchymal stem cells-engineered system enhances the therapeutic effects altering tumor microenvironment

**DOI:** 10.1186/s12985-020-01444-5

**Published:** 2020-11-13

**Authors:** Mohsen Keshavarz, Mir Saeed Ebrahimzadeh, Seyed Mohammad Miri, Hassan Dianat-Moghadam, Seyedeh Sara Ghorbanhosseini, Seyed Reza Mohebbi, Hossein Keyvani, Amir Ghaemi

**Affiliations:** 1 The Persian Gulf Tropical Medicine Research Center, The Persian Gulf Biomedical Sciences Research Institute, Bushehr University of Medical Sciences, Bushehr, Iran; 2 Department of Medical Virology, School of Medicine, Iran University of Medical Sciences, Tehran, Iran; 3 Department of Microbiology, Golestan University of Medical Sciences, Gorgan, Iran; 4 Department of Virology, Pasteur Institute of Iran, Tehran, Iran; 5 Stem Cell Research Center, Tabriz University of Medical Science, Tabriz, Iran; 6 Department of Clinical Biochemistry, Faculty of Pharmacy, Isfahan University of Medical sciences, Isfahan, Iran; 7 Gastroenterology and Liver Diseases Research Center,Research Institute for Gastroenterology and Liver Diseases, Shahid Beheshti University of Medical Sciences, Tehran, Iran

## Correction to: Virology Journal (2020) 17:64 https://doi.org/10.1186/s12985-020-01326-w

Following publication of the original article [1], we have been informed that due to an oversight, the affiliation of author Seyed Mohammad Miri has been presented incorrectly.

The incorrect affiliation currently reads:

Department of Chemistry, Sharif University of Technology, Tehran, Iran

The correct affiliation should read:

Department of Virology, Pasteur Institute of Iran, Tehran, Iran

The original article has been corrected.

## References

[1] Keshavarz (2020). Virology Journal.

